# An Investigation on the Influence of Operation Experience on Virtual Hazard Perception Using Wearable Eye Tracking Technology

**DOI:** 10.3390/s22145115

**Published:** 2022-07-07

**Authors:** Siyu Li, Yongqing Jiang, Chao Sun, Kangkang Guo, Xin Wang

**Affiliations:** School of Measurement and Communication Engineering, Harbin University of Science and Technology, Harbin 150080, China; lsyddyx@163.com (S.L.); jiangyongqing@126.com (Y.J.); wxin08042@163.com (X.W.)

**Keywords:** eye-tracking, visual search, electrical safety, hazard recognition, hazard perception, experience level, cognitive

## Abstract

Poor electrical hazard recognition is a widespread issue in the production industry. Hazard perception has impacts workers’ hazard recognition, causing them to experience unanticipated hazard exposure and suffer catastrophic injuries. To improve the factors of affecting hazard perception, the current study examined hazard recognition as an everyday visual search task. A comparative test was carried out combining the advantages and disadvantages of the two test methods. It was confirmed that the virtual image test data can replace the real image test data and demonstrate superior flexible settings performance, so the virtual image test method is used. A hazard perception test method based on wearable eye tracking technology was proposed to analyze the eye-tracking data (i.e., fixation, count, search duration, mean fixation duration, eye tracking, and hazard recognition performance feedback) were compared between experts in the field of electrical safety: skilled workers with at least five years of work experience and workers who had been on the job for less than a year. It was found that experts had a better hazard recognition accuracy and missed detection rate than other workers. Experts’ hazards research track was more concised and paid less attention time. This advantage is most obvious in complex risk environments. The findings also suggest that workers who have different working years was not obvious visual search patterns other than the search duration. As can be seen the work experience is not an absolute factor in improving hazard perception. The present research will be useful to understand the influence of working years on hazard perception and provide a theoretical basis for corresponding training.

## 1. Introduction

Hazard recognition is generally regarded as the first and most fundamental element of any injury prevention program [1]. When hazards remain either unrecognized or unmanaged, catastrophic and unanticipated injuries can follow [2]. To improve hazard recognition levels, a number of interventions (e.g., job-hazard analysis, safety training, and safety checklists) have been adopted in practice [3]. However, desirable levels of hazard recognition have not been achieved due to weaknesses in traditional interventions [4]. In fact, recent research has demonstrated that several interventions are designed without a proper understanding of the hazard recognition process [5]. Hazard recognition is largely a visual search task [6], and workers do not pay attention and deliberately fail to follow safety codes, mainly due to poor hazard perception [7]. Electric work belongs to the category of special operations; it has the characteristics of high hazard and frequent accidents [8]. Electric work needs higher requirements for hazard perception. Due to the difference in hazard perception, many hazards cannot be effectively identified, resulting in accidents [9]. Studying the differences in electrician hazard perception has important implications for training [10] and safe operation.

Hazard perception is a complex and multi-faceted concept. Many studies have found that experience has a certain impact on it [11]. In terms of driving, Borowsky, Vinson, et al. [12,13] analyzed the influence of driving age on hazard perception and found that novice drivers have poor hazard perception and slower response to potential hazards. Sun [14] and Upahita D P [15] analyzed the influence of driving experience on the driver’s hazard perception process and found that experienced drivers were significantly better than inexperienced drivers in terms of hazard reaction time, hazard identification, and hazard assessment. In the construction field, Hossein Karim et al. [16] examined the effect of the experience of construction workers on reducing the consequences of human error. In most accidents, higher experience greatly reduces the fatality rate. Liu [17] found that the coincidence of the actual flight trajectory of the experienced flight instructor with the predetermined flight trajectory in the experiment was better than that of the inexperienced flight student. These studies show that in many fields, different levels of experience can make differences in hazard perception. At present, the research on the experience impact of special operators is relatively scarce.

In recent years, eye-tracking technology has been used in different industries [18], especially high hazard work, to assess the current and future work environment. For example, eye tracking is used in medical surgery, aviation, driving, lifting operations, and maritime industries to improve teaching strategies and student performance. It also can provide a scientific and rational decision-making basis for safe operations [19,20,21,22,23,24,25]. To improve safety, eye-tracking has also been used to understand hazard perception and hazard identification for construction and confined space operations [26,27].

This study, examining hazard recognition as an everyday visual search task, analyzed the eye-tracking data in the field of electrical safety. The present research will be useful to understand the influence of working years on hazard perception and provide a theoretical basis for corresponding training.

## 2. Study Protocol

### Experimental System Construction

**Figure 1:**
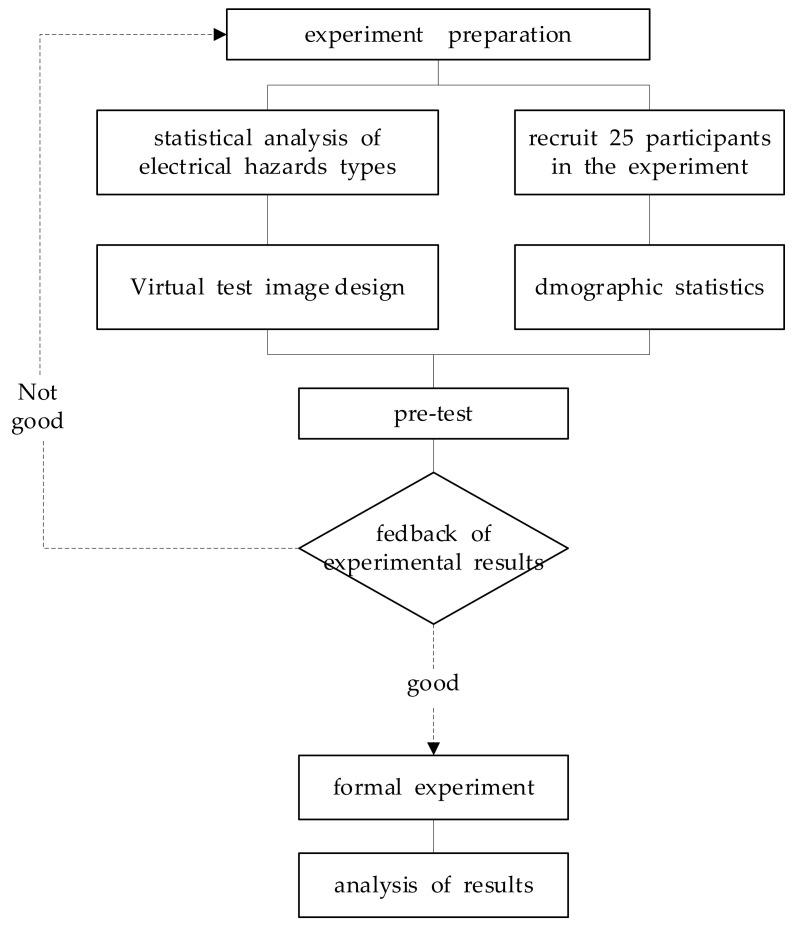
Experimental framework.

In order to determine the characteristics and differences in hazard perception of electrical workers with different experience levels, we designed a 3 (experience: A1 expert, A2 experienced, A3 inexperience) × 3 (virtual hazard case images: H1, H2, H3) experiment. The hazard recognition activity used hazard case images’ information that were captured from a panel of seven safety professionals in a previous study and represented a variety of types of incidents for electrical injuries including electric shock, fire, explosion, and others. The workers were tasked with identifying all safety hazards in each of the case images verbally. All participating workers’ eye-tracking data including fixation time and eye track path was gathered. The experimental framework is shown in Figure 1.

(1) In order to obtain the valid hazard perception data of an electrical worker, we conducted two experimental methods to compare: the real case image test and the virtual case image. The more suitable experimental method was selected by comparing the experimental results.

**Figure 2:**
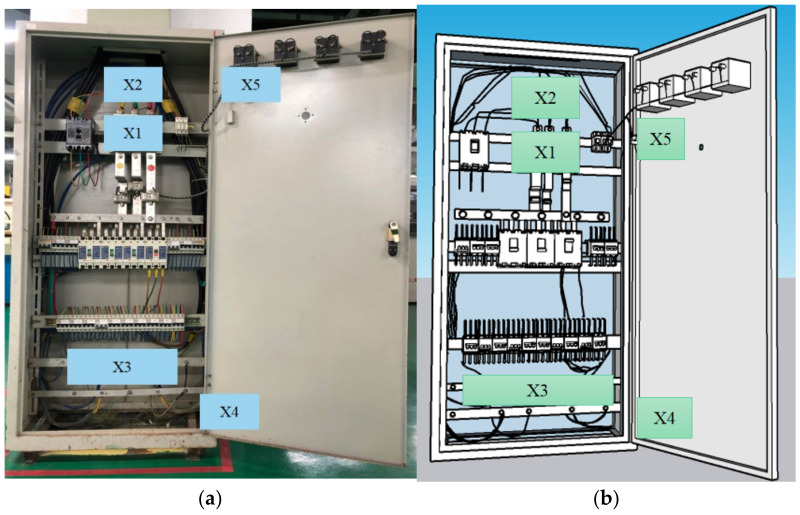
Hazard distribution area. (**a**) real scene, (**b**) image test.

We used a picture that was captured from a switchboard located in an engineering training center as the real case test image. It included five safety hazards. The virtual case image used the Sketch Up 19.0.685 software (Trimble, Sunnyvale, CA, USA) to draw the test image based on the real case test image. The hazard information settings are shown in Table 1, and the experimental materials of the two test methods are shown in Figure 2. A total of twelve workers were recruited and divided into two groups to participate in two forms of hazard perception test experiments.

The workers were asked to participate in this hazard perception pre-experience while their visual search patterns were captured using an eye-tracking device. One group of invalid data was excluded. A total of 11 sets of data were obtained.

Compare the above two experimental data; the results are shown in Table 2.

SPSS26.0 software(IBM, Armonk, NY, USA) was used to compare and analyze the fixation count, saccade count, and hazards recognition accuracy. The results are shown in Table 3. An LSD-test was used to discuss whether there were significantly differences in the data between the two experiments. The results presented no significant differences (i.e., *p* < 0.05); it indicated the virtual image test data can replace the real image test data and demonstrated superior flexible settings performance, so the virtual image test method is used.

**Figure 3:**
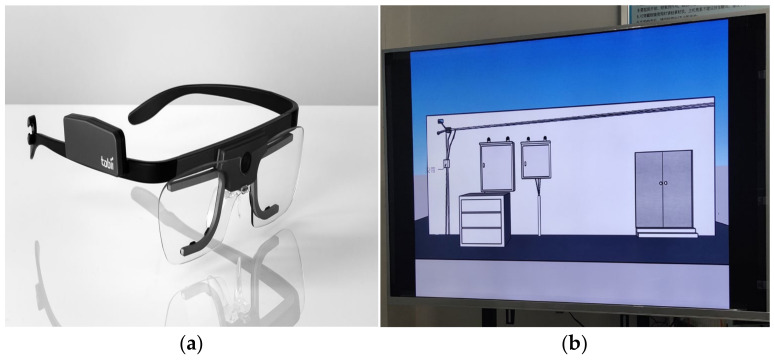
Laboratory equipment. (**a**) Tobii Glasses 2 Wearable Eye Tracker, (**b**) Experimental Electronic Display Screen.

(2) The experimental equipment is shown in Figure 3. The Tobii Glasses 2 wearable eye tracker(KiNGFAR, Beijing, China) was used, with a tracking frequency of 50 Hz, to track eye movements using the principles of corneal reflection, binocular acquisition, and dark pupil tracking (Figure 3a). In terms of application programs, Tobii Glasses Controller control software (KiNGFAR, Beijing, China) and ErgoLAB human–machine environment test cloud platform were used to collect experimental data and analyze and process data.

The platform collects and provides a variety of visual search patterns (e.g., search duration, fixation time ratio, fixation count, eye-tracking Information Heatmap, eye movement information track map, etc.). It achieves eye-tracking visual attention feedback and reflects subjective psychology through objective eye movement data.

The experimental scene was played on a 52-inch electronic display screen (Figure 3b).

**Figure 4:**
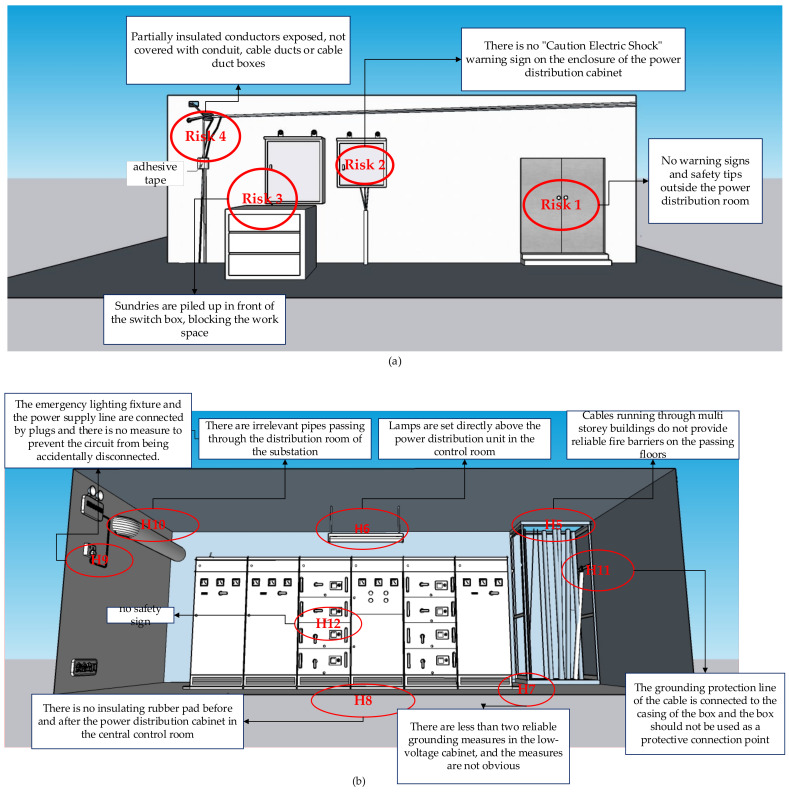

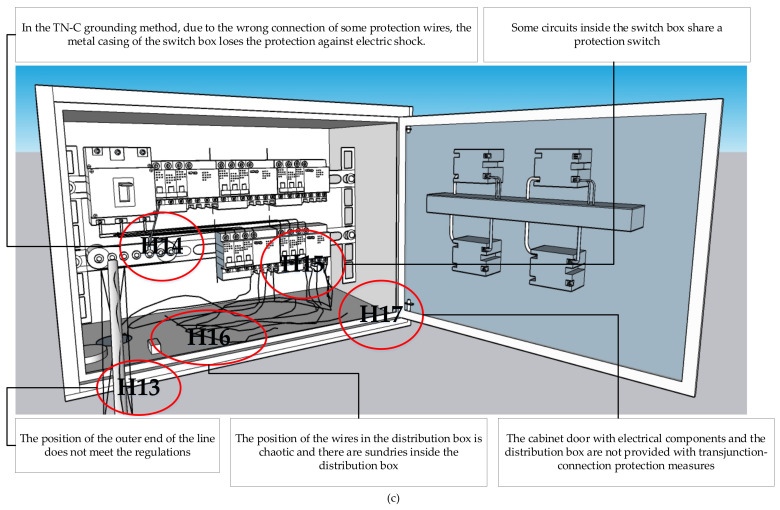
Design drawings of three test images. (**a**) Test image 1, (**b**) Test image 2, (**c**) Test image 3.

(3) To summarize the causes of electrical accidents in recent years and the actual situation of electrical hazard inspections of many companies, we designed a variety of hazards including electric shock, fire, explosion, and others. There are a total of three virtual case images as test images, including seventeen safety hazards. Among them, test image 2 and 3 have more hazard information, densely arranged, and more complex scene settings. The setting of hazard factors and the description of hazard information are shown in Figure 4. (Note: The test images observed by the workers were the versions marked with no hazard information).

(4) Selection of experimental indicators. Fixation and saccade are two main eye movement behaviors. Fixation refers to when the eye movement behavior stays in a fixed area for a short time. The acquisition of information is mainly completed through the fixation behavior, and the position of the fixation represents the focus of a person’s attention. Saccade is a rapid and sudden eye movement that separates the fixation point and indicates the direction of the eye’s focus as the eye shifts from one fixation point to another. During fast movement, no information can be obtained.

During the experiment, the hazard recognition and search duration of participating workers was recorded in each test image. The differences in the hazard perception results of different experience levels can be seen. The selected experiment indicators are shown in Table 4.

**Figure 5:**
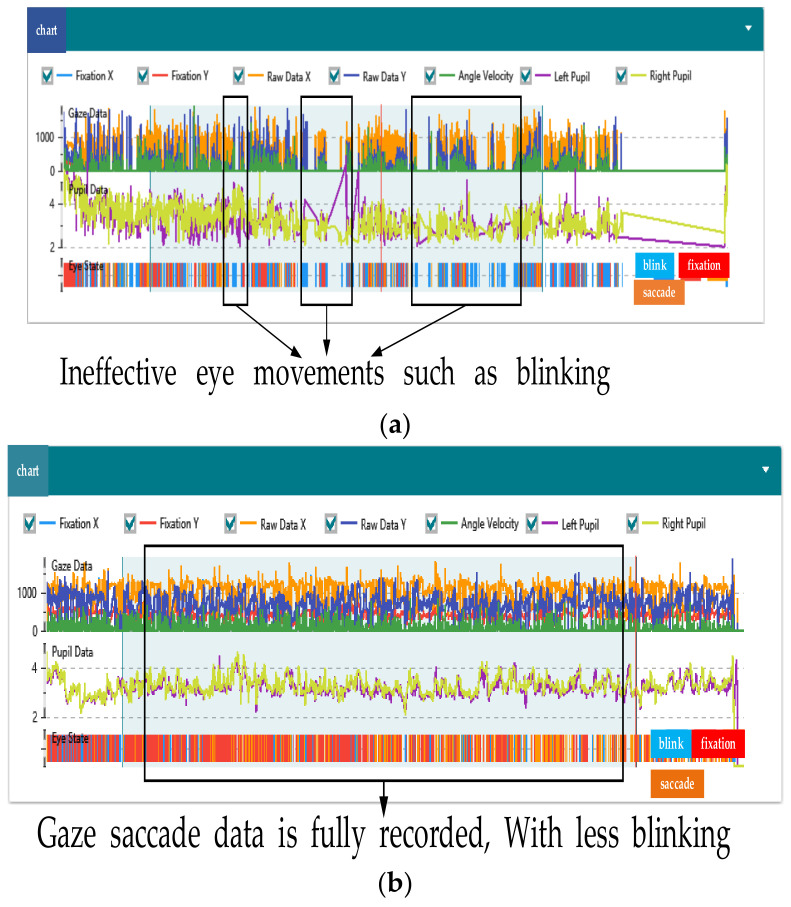
Valid and invalid data experiment results. (**a**) Invalid data sample, (**b**) valid data sample.

(5) Worker sample. The workers showed a large number of eye movement features during the experiment, but due to the limitation of the sensitivity of the equipment, some eye movement features could not be recorded. At the same time, during the search process, the workers also experienced excessive eye movement, frequent blinking, squinting, and other behaviors, as well as visual fatigue caused by the length time of the experiment. These factors all affect the validity of the data. To accomplish the research objectives, the eye data with validity of less than 80% were excluded, as shown in Figure 5a, as invalid data samples. It can be seen that the workers had more blinking behaviors during the experiment, resulting in some data not been recorded, leaving many vacancies. Figure 5b is a valid data sample. During the experiment, the data were recorded comprehensively, with more gaze and saccade behaviors, and less blinking, which had no effect on the overall data. After screening, 25 groups of valid workers’ data were retained.

A convenient sample of 25 electrical works was recruited from internal workers when collecting test image information. The workers were divided into three groups according to their work experience levels, as shown in Table 5. All the selected workers had no ocular disease and had normal or corrected-to-normal vision. Given that empirical studies of this nature provide the opportunity to collect multiple data points per trial individual, smaller sample sizes are appropriate, relative to larger survey-style studies. This study satisfies the sample size requirements discussed by Pernice and Nielsen, who specify that sample sizes in eye-tracking studies vary widely, ranging from 6 to 30.

## 3. Experimental Procedure and Data Acquisition

### Experiment Process

The experiment involved three main testers; one was responsible for the operation of the eye tracking device, the other was responsible for providing necessary explanations to the workers and recording the hazard recognition of the workers during the experiment, and the other main tester was responsible for inviting the participants, who completed the experiment in the hazard recognition task, to answer some questions. In order to avoid random errors in the experiment, the three main testers remained unchanged throughout the experiment.

In order to test the effect of the experiment, a pre-experiment was carried out before the formal experiment. Through the pre-experiment, it was found that when using a computer screen to play a test image, the data of some fixation points were inaccurate due to the small screen. Therefore, in the formal experiment, a 52-inch display screen was used to play the test images.

Before the formal experiment began, a short survey was required to ask participants about their demographic information (e.g., age, gender, education level, years of work, certificates and training obtained, etc.). The workers were required to complete the instrument calibration in order to accurately record their eye movement data during the experiment. The calibration method is achieved by the worker wearing the eye-tracking device and the fixation point being completely coincident with the designated position. If they failed several times, the worker was rejected from participating in the experiment.

**Figure 6:**
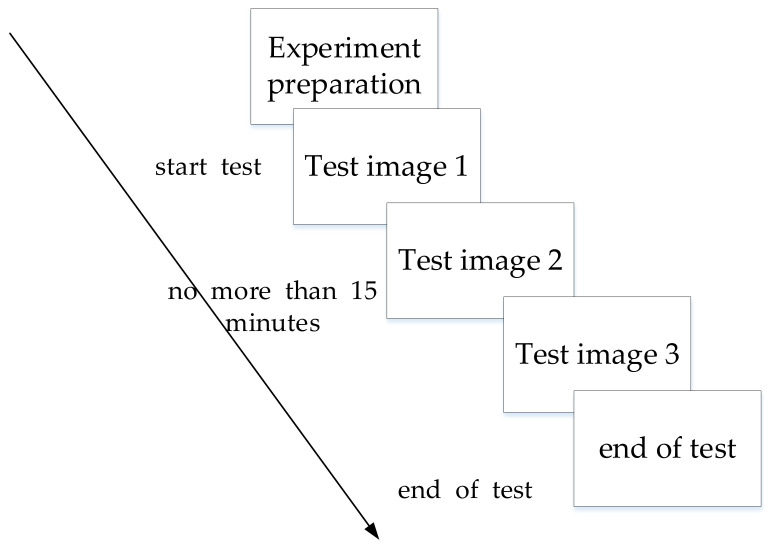
Hazard perception test flow chart.

The experimental test flow is shown in Figure 6. More specifically, the participating workers stood before a screen at a distance of 1 m and observed three test images in turn, with the observation order of the three images being fixed. After one worker completes all tests, the next continues. The workers were tasked with identifying all safety hazards in each of the test images verbally. The experiment’s total time should not exceed 15 min. If the workers pointed out the hazard of the target and correctly explained the reason why the target poses a hazard, it was marked as a successful identification. For example, when workers identified hazard 2 in test image 1 and explained that there was no safety warning sign attached to the power distribution cabinet, we regarded it as a correct identification, and any other explanation would be regarded as a misjudgment. If workers pointed out another area, but provided a reasonable explanation for it, the situation was not considered a false positive. For example, workers regarded the sign (safe area) at the safety exit in the lower left corner of the test image 2 as a hazard and suggested that the completeness of the sign should be checked, even though there may have been a sign in the image. In addition, due to viewing angle issues, the installation location of the safety sign was deemed to be unsatisfactory; such an identification would not be considered as a misidentification. When necessary, the examiner could provide the workers with further information about the type of operation, materials, equipment used, etc.

After the test was completed, the examiner helped the workers to remove the experimental equipment and communicated with the workers to understand how they searched for and identified potential hazard during the experiment.

## 4. Analysis of Results

### 4.1. Analysis of Risk Perception Results

The hazard recognition accuracy (AHR) of the participating workers for three test images was calculated as proportion of hazards identified (expressed as a percentage) using Equation (1).

(1) $$\mathrm{AHR}=\frac{\mathrm{CHR}}{3{\mathrm{HR}}_{i}}$$

where CHR represents the number of correctly identified hazards and $3{\mathrm{HR}}_{i}$ represents the total number of hazards in three test images as judged by the worker.

The missed detection rate (MHR) of the participating workers for three test images was calculated as a proportion of hazards unidentified (expressed as a percentage) using Equation (2).

(2) $$\mathrm{MHR}=\frac{3\mathrm{HR}-\mathrm{CHR}}{3\mathrm{HR}}$$

where 3HR represents the total number of hazards in three test images.

The fixation time included the search duration and mean fixation duration. The mean fixation duration of the participating works for three test images was calculated as the average of total correctly identified hazards’ fixation time.

**Figure 7:**
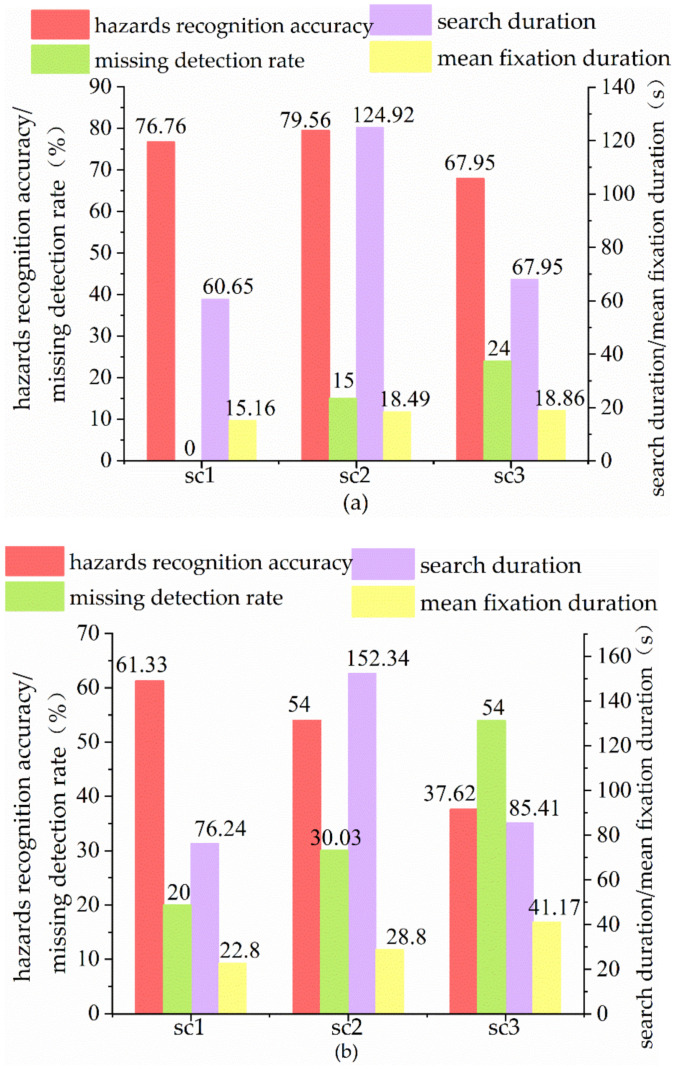

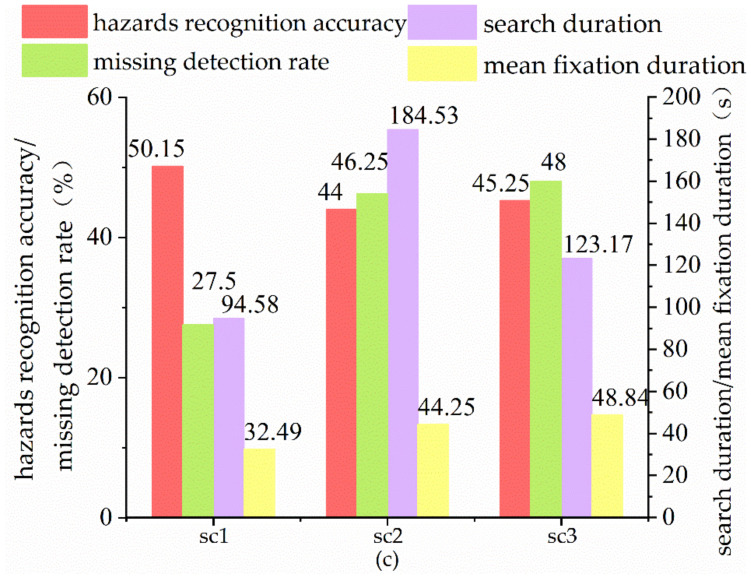
Statistical chart of hazard recognition of three groups. (**a**) Hazard recognition of group A1, (**b**) Hazard recognition of group A2, (**c**) Hazard recognition of group A3.

As can be seen from Figure 7, in hazard recognition accuracy metric (AHR), the group A1 has the highest data in test image 2 and the groups A2 and A3 have the highest data in test image 1. The groups A1 and A3 have the lowest data in test image 3 and the group A2 has the lowest data in test image 2.

In missed detection rate metric (MHR), the three groups showed a similar trend of data growth and with the increase in the complexity of the test images, the missed detection rate gradually increased. In test image 1, group 1 found all hazards. Even in the most difficult test image 3, the experts still showed over 67% hazards recognition accuracy. It shows that the experts demonstrated superior performance in hazard identification, and high difficulty hazard identification is the main indicator to measure the difference between experts and other workers. In the process of raising a novice into an expert, it is also necessary to continuously strengthen the training on difficult hazards.

In search duration metric, the three groups spent the most time in searching for test image 2 and mean fixation duration in test image 3. The three groups spent the least search duration and mean fixation duration in test image 1. It shows that with the increase in hazard difficulty, people with different work experience all needed more fixation time for hazard recognition.

In order to further compare the difference in hazard perception of the three groups, the groups were compared using ANOVA and test-LSD for hazard perception metrics (hazards recognition accuracy, missed detection rate, search duration, and mean fixation duration). After calculation, the indicators met the analysis requirements. The results are shown in the Table 6.

**Figure 8:**
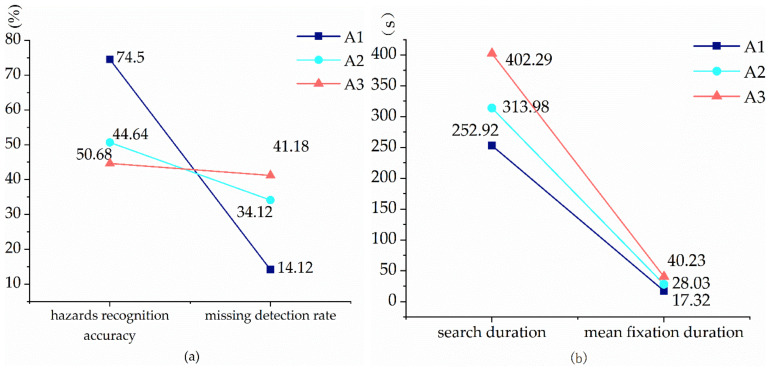
Comparison chart of hazard recognition data for three groups of workers. (**a**) Comparison of hazards recognition accuracy and missed detection rate of workers. (**b**) Comparison of the search duration and mean fixation duration of the workers’ hazard search.

The following sections discuss the results of the testing. As can be seen from Table 6 and Figure 8, the experts are significantly better than experienced and inexperienced workers in four metrics of hazard perception. The three groups are significantly different from each other in missed detection rate, search duration, and mean fixation duration than other groups (PA1-A2 = 0 < 0.05, PA1-A3 < 0.05, PA2-A3 < 0.05). The data superiority of the three groups decreases sequentially and the index superiority of the A1 group is the best. In the hazards recognition accuracy metric, the expert has significant differences than other groups (*p* < 0.001). There is an insignificant difference between the other two groups. It shows that experience factors are not the primary reasons affecting the hazard perception of electrical workers; experienced electrical workers are not necessarily more hazard aware than inexperienced electrical workers. The years of work experience do not necessarily help electrical workers recognize hazards, and general work experience is not equivalent to specific safety experience, i.e., it does not necessarily improve the hazard perception of operators.

### 4.2. Analysis of the Difference in Thefixation Count

**Figure 9:**
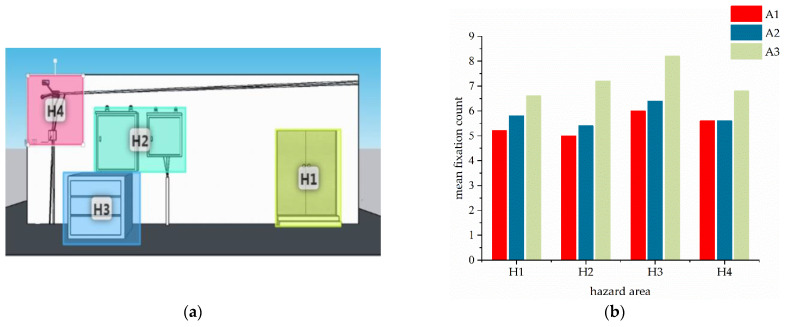

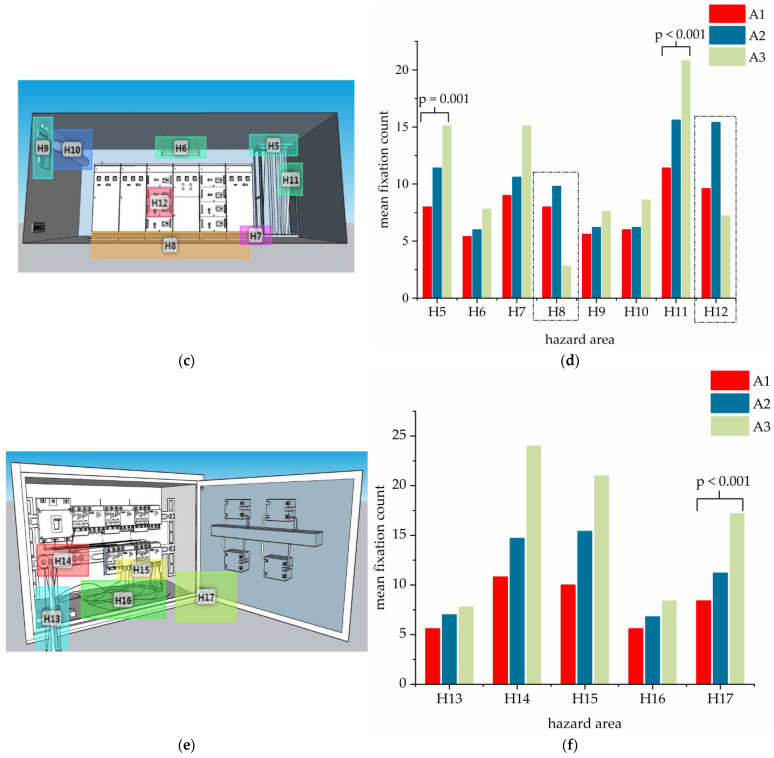
Statistical graph of hazard distribution and mean fixation count in the test images. (**a**) AOI division in test image 1, (**b**) fixation count in test image 1, (**c**) AOI division in test image 2, (**d**) fixation count in test image 2, (**e**) AOI division in test image 3, (**f**) fixation count in test image 3. Boxes in Figure 9d represent abnormal data. Except for the two hazard areas, H8 and H12, in test image 2, the A3 group had higher attention than the workers in the A1 and A2 groups on the remaining hazard area.

The area of AOI (interest area–hazard area) is divided in the test image. The mean fixation count in AOI was shown in Figure 9 and Table 7.

As can be seen from Table 7, except for the two hazard areas, H8 and H12, in test image 2, the A3 group had higher attention than the workers in the A1 and A2 groups on the remaining hazard area. There were considerable data on the mean fixation count metric of H14 and H15, showing that the workers need to spend more time to recognize hazards and that the hazards are difficult to judge. For most hazards, inexperienced workers have higher mean fixation count than experienced workers and experts. It indicates that, in the process of hazard identification, inexperienced workers are less confident in determining hazard information than experienced workers. Experienced workers often make decisions earlier and can identify complex and hidden hazards in less time. There are different phenomena in H8 and H12: inexperienced workers have less mean fixation count than experienced workers. Through interviews, we learned the reason for this phenomenon is that most inexperienced workers do not notice the hazards. It shows that the two hazard areas are easily ignored by inexperienced workers.

In order to further compare the difference in hazard area of the three groups, group comparison using ANOVA and test-LSD for mean fixation count was performed. After calculation, all the hazard area indicators met the analysis requirements (ANOVA *p* < 0.05), except for H1, H3, and H15. The results are shown in Table 8.

The analysis of test-LSD showed that the three groups are significantly different from each other in H5, H11, H14, and H17. The A1 group and A3 group are significantly different from each other in H2, H6, H7, H8, H9, H10, and H16. The A1 group and A2 group are significantly different from each other in H12. It shows that there are significant differences between experts and other workers in fixation count. However, there was no significant difference between inexperienced and experienced workers, indicating that years of experience had no significant effect on the fixation count.

### 4.3. Typical Scanning Path Analysis

**Figure 10:**
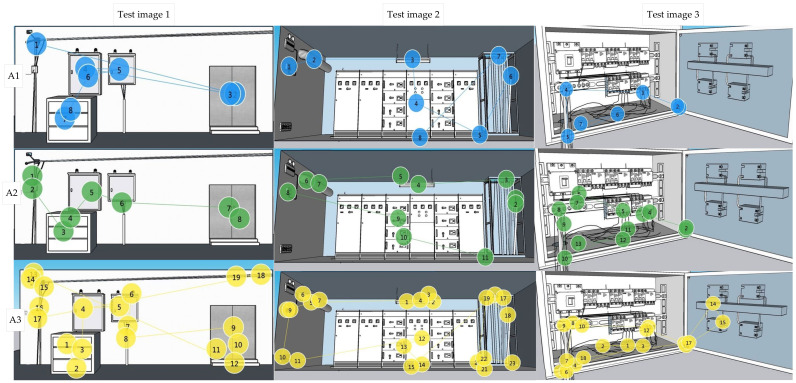
The typical scanning path of workers in three test images. A1 represents A1 group (electrical safety experts), A2 represents A2 group (skilled workers with at least five years of work experience), A3 represents A3 group (employees with less than a year on the job).

The eye movement heat map formed by the overall data of each group of workers is compared with the sequential records of workers’ hazard recognition during the experiment, and finally, a typical scanning path is formed. The scanning paths of the three group workers’ hazard recognition processes are shown in Figure 10. The hazards in all test images can be divided into two types: equipment hazards (e.g., H4, H7, H11, etc.) and environmental hazards (e.g., H1, H3, H16, etc.). From the scanning paths of the three groups of workers, it can be seen that the scanning paths of the A1 group are similar to A2 group, and they are usually checked in the order from the device to the environment. However, the A3 group is the opposite; they prioritized checking the environment and then checking the equipment related hazards. The complexity of the scanning paths of the three groups gradually increased and the A1 group was the most concise.

The first noticed hazards in the three test images of group A1 were H4, H9, and H17. All were line or grounding problems which could easily lead to the hazard of major accidents. It shows that experts will first evaluate high hazard targets in the process of hazard recognition. The scanning path of the A3 group is not as regular as the A1 and A2 groups because search sequence is often disrupted due to other factors in the environment; the same hazard area is repeatedly checked and more attention paid to safety areas.

Compared with the systematic inspection process of experts, the experienced workers and inexperienced workers were more likely to recognized hazards according to their subjective wishes and were easily affected by the environment and equipment during the recognition process. Safety areas in complex environments can distract both experienced workers and inexperienced workers.

## 5. Conclusions

The experts demonstrated superior performance in hazard perception metrics (i.e., search duration, mean fixation duration, hazards recognition accuracy, missed detection rate) compared to others. High difficulty hazard identification is the main indicator to measure the difference between experts and other workers. In the process of raising a novice into an expert, it is also necessary to continuously strengthen the training on difficult hazards. Experienced workers and inexperienced workers have significant differences in the other three metrics, except hazard recognition accuracy. The years of work experience do not necessarily help electrical workers recognize hazard, and general work experience is not equivalent to specific safety experience, i.e., it does not necessarily improve the hazard perception of operators.

For most hazards, inexperienced workers have a higher mean fixation count than experienced workers and experts. In the process of hazard identification, inexperienced workers are less confident in determining hazard information than experienced workers. Experienced workers often make decisions earlier and can identify complex and hidden hazards in less time. Most inexperienced workers easily ignore hazards. There was no significant difference between inexperienced and experienced workers, and the years of experience had no significant effect on the fixation count.

In scanning paths, the experts first evaluated high hazard targets in the process of hazard recognition and usually checked in the order from the device to the environment. Compared with the systematic inspection process of experts, the experienced workers and inexperienced workers were more likely to recognize hazards according to their subjective wishes and were easily affected by the environment and equipment during the recognition process. Safety areas in complex environments can distract both experienced workers and inexperienced workers.

This research innovatively explores operation experience’s influence on hazard perception and provides a theoretical basis and research direction for how subjective factors affect hazard perception. On this basis, we will continue to explore ways to adopt personality intervention to improve hazard perception, since the follow-up research will be compared with accessing hazardous sites for dynamic testing. In order to maintain the consistency of the equipment the research, we used wearable eye tracking technology.

## Figures and Tables

**Table 1:** Hazard information description.

Hazard Area	Hazard Description
X1	Main switch damaged
X2	Damaged wire insulation
X3	Poor contact on the bottom line
X4	The box is not grounded for protection
X5	Damaged voltmeter

**Table 2:** Comparison of Test Methods for Hazard Perception of Electrical Workers.

Method	Disadvantage	Advantage
Real scene test	More consideration for safety in experiments. The scene cannot be changed. Has many uncertain interference factors (noise, debris, people). The data obtained has certain limitations. Experiments are limited by the environment.	Demonstrate the authenticity of the risk perception test. The results obtained are highly accurate. Better interactivity.
Image test	There is a gap with the real scene. Scene recognition is limited.	Simple and easy to operate, the experiment is not limited by the site. Risk factors can be designed in a unified manner, and scenarios can be arbitrarily established as needed. You can change the scene settings for multiple experiments and comparison experiments. Meets the needs of differentiated research conditions, and it is easy to set influencing factors such as noise and location.

**Table 3:** Analysis of experimental data difference between two hazard perception test methods.

Data Metrics	Mean Square	F	P
fixation count	280.084	0.154	0.704
saccade count	17,673.409	1.862	0.206
hazards recognition accuracy	0.012	0.511	0.493

**Table 4:** Experimental metrics and instructions.

Experimental Metrics	Metric Description
Hazards recognition accuracy	The percentage of correctly identified hazardsin the total number of identified hazards.
Missing detection rate	The percentage of unidentified hazard in the total number of targeted hazards.
Search duration	Total time (s) spent searching for each test image.
Mean fixation duration	Average time (s) to successfully recognized the hazards.
Fixation count	The number of workers’ fixation in each area of interest.
Eye track path	The eye movement path formed by the workers’ observation of stimulus information.

**Table 5:** Explanation of the division of experimental subjects.

Group	Experience Level	Subject
A1	Experts in electrical safety field.	5 people
A2	Experienced operators (such as electricians or safety managers) working in electrical places, with at least five years of work experienceand corresponding qualification certificates.	10 people
A3	Those who have obtained the qualification certificate of electrician, have not officially joined the work or have worked for less than one year.	10 people

**Table 6:** Comparison of differences in hazard recognition results among three groups of workers.

			Hazards Recognition Accuracy	Missing Detection Rate	Search Duration	Mean Fixation Duration
Experience level		F	21.983	31.575	78.427	77.444
		P	<0.001	<0.001	<0.001	<0.001
LSD	A1-A2	SE	4.354	3.421	12.620	1.914
		P	<0.001	<0.001	<0.001	<0.001
	A1-A3	SE	4.354	3.421	12.620	1.914
		P	<0.001	<0.001	<0.001	<0.001
	A2-A3	SE	3.555	2.794	10.304	1.563
		P	0.230	0.019	<0.001	<0.001

**Table 7:** The mean fixation count of three groups at each hazard area.

	Hazard Area							
Test image 1	H1	H2	H3	H4				
A1	05.20	05.00	06.00	05.60				
A2	05.80	05.40	06.40	05.60				
A3	06.60	07.20	08.20	06.80				
Test image 2	H5	H6	H7	H8	H9	H10	H11	H12
A1	08.00	05.40	09.00	08.00	05.60	6.00	11.40	09.60
A2	11.40	06.00	10.60	09.80	06.20	6.20	15.60	15.40
A3	15.10	07.80	15.10	02.80 *	07.60	8.60	20.80	07.20 *
Test image 3	H13	H14	H15	H16	H17			
A1	05.60	10.80	10.00	05.60	08.40			
A2	07.00	14.70	15.40	06.80	11.20			
A3	07.80	24.00	21.00	08.40	17.20			

* represents abnormal data. Except for the two hazard areas, H8 and H12, in test image 2, the A3 group had higher attention than the workers in the A1 and A2 groups on the remaining hazard area.

**Table 8:** The test-LSD for mean fixation count.

Hazard Area (AOI)	P A1–A2	A2–A3	A1–A3
A1–A2–A3			
H5	0.008	0.001	0.000
H11	0.000	0.000	0.000
H14	0.005	0.000	0.000
H17	0.014	0.000	0.000
A1–A2, A3			
H2	0.602	0.008	0.008
H6	0.393	0.004	0.002
H7	0.104	0.000	0.000
H8	0.097	0.000	0.000
H9	0.453	0.040	0.018
H10	0.832	0.005	0.011
H16	0.157	0.026	0.002
Others			
H12	0.015	0.015	0.289
H4	1.000	0.092	0.165
H13	0.180	0.343	0.004

## Data Availability

Data is available in the paper.
